# Investigating the Benefits of Tectonite Dust as an Amendment for Bark Substrates and Dryland Crops

**DOI:** 10.3390/plants13010126

**Published:** 2024-01-02

**Authors:** Lloyd Nackley, Luke Van Lehman, Owen Van Lehman, James S. Owen, Carolyn Scagel

**Affiliations:** 1 Department of Horticulture, Oregon State University, Corvallis, OR 97333, USA; 2 North Willamette Research and Extension Center, Oregon State University, Aurora, OR 97002, USA; 3 Application Technology Research Unit, Agricultural Research Service, U.S. Department of Agriculture, 1680 Madison Ave., Wooster, OH 44691, USA; jim.owen@usda.gov; 4 Horticultural Crops Production and Genetic Improvement Research Unit, Agricultural Research Service, U.S. Department of Agriculture, 3420 NW Orchard Avenue, Corvallis, OR 97330, USA; carolyn.scagel@usda.gov

**Keywords:** dryland farming, mineral waste, Oregon, soilless substrates, sustainable farming, waste utilization

## Abstract

This study investigates the potential benefits of using tectonite dust as a soil amendment in central Oregon. Tectonite, a rare mineral byproduct of the Warm Springs Composite Products Company, has unique properties that can enhance soil fertility and water-holding capacity. The study includes analyses of tectonite’s physical and chemical properties, small-scale growth trials, and farm-scale experiments to measure grain yield. Physical property analysis demonstrated that tectonite increased water-holding capacity and improved soil structure when added to bark substrates. Responses varied in mineral soils, affecting air space, and water-holding capacity. Small-scale trials showed positive growth responses in wheat height and biomass, indicating improved early growth and establishment. Farm-scale experiments confirmed increased grain yields with tectonite application. These findings suggest that tectonite enhances soil health and crop yields by improving structure, nutrient availability, and water retention. Careful sourcing and testing are necessary to address potential heavy metal contamination risks. Using tectonite as a soil amendment aligns with sustainability goals, reducing waste, and greenhouse gas emissions. It may also offer cost savings compared to synthetic fertilizers and stimulate the local economy. Further research is needed to understand the long-term effects of tectonite on edible crops and heavy metal content. Nevertheless, tectonite shows promise as a sustainable soil amendment for promoting agriculture in central Oregon. By exploring its potential benefits, farmers can enhance soil fertility, improve water-use efficiency, and contribute to a more sustainable agricultural system. This study highlights the importance of utilizing waste byproducts in agriculture to achieve environmental and economic sustainability. Tectonite has the potential to play a significant role in addressing water scarcity and enhancing crop productivity in arid regions like central Oregon.

## 1. Introduction

Farmers in central Oregon are facing increasingly arid conditions, which are forcing them to consider alternative strategies to ensure efficient uptake of limited soil water provided by an average 223 mm of annual precipitation. Improving crop water-use efficiency is essential for the millions of acres of dry land wheat grown in the northwestern US [1]. At the same time, the Warm Springs Composite Products Company (WSCP) creates thousands of tons of mineral waste material when shaping raw tectonite into fire-proof door cores. This creates an opportunity to divert their by-product stream into a potentially beneficial agricultural soil amendment. The unutilized byproduct is dust of a mineral structure known as tectonite. Tectonite is a rare mineral that belongs to the pyroxene group. It is typically found in metamorphic rocks, particularly in regions that have undergone high levels of deformation due to tectonic activity [2]. Tectonite is known for its distinctive properties, such as its blue-green color, strong cleavage, and high density. Its crystal structure is characterized by tightly packed chains of silicate tetrahedra, which give the mineral its unique properties. WSCP first used tectonite in 1993. Three decades later there is a need to develop a transformative waste stream to shift the costly and energy-inefficient disposal of under-utilized tectonite by-product to distant landfills. WSCP became interested in diverting tectonite towards agriculture due to the similar physical and chemical properties of gypsum, which is widely used in central Oregon as a soil amendment to increase water infiltration of naturally occurring acidic soils. If tectonite could become a viable soil amendment, WSCP would have the opportunity to reduce disposal costs, enhance soil fertility, and create a new market opportunity for the Confederated Tribes of Warm Springs, which is one of the most impoverished areas in the United States [3,4].

The WSCP is situated in central Oregon, surrounded by a mix of forestry, range land, and conventional agricultural crops. The region boasts 65,000 irrigated hectares across the surrounding three counties of Deschutes, Crook, and Jefferson, where potatoes, alfalfa, and other hay crops are grown on a commercial basis. Additionally, there are thousands of hectares of dry land used for forage and cereal crops, which rely on limited natural precipitation [5]. The challenge of producing the forage and cereal crops has been exacerbated by extreme drought. These worsened dry seasons result in water scarcity, planting delays, increased wildfire risk, decreased pool depth in reservoirs and lakes, and an increasing need for routine irrigation throughout the season. Adding amendments such as organic matter and biochar to soils is a common method to increase the water-holding capacity of the soil [6,7,8]. Fine particle mineral soil amendments are also used to increase soil aggregation, which results in a shift from macro- to micro-soil-porosity and subsequent increase in soil water-holding capacity, soil fertility, and crop growth [9]. It is currently unknown if tectonite could improve soil water-holding capacity for conventional and specialty crop farmers in the surrounding arid region and northwest US region.

Similarly, the addition of 8% to 10% by vol of temperature pre-treated industrial clay aggregates has been seen to increase water holding capacity of pine bark-based soilless substrates [10]. Similarly, to soils, lime, dolomite, or gypsum are added to soilless culture to increase and maintain pore water pH, increase availability of calcium and magnesium while providing a pH buffer, or provide plant available calcium and sulfur, respectively [11]. Thus, this provides another marketplace for the byproduct to be used. Other than southern California, the Willamette Valley greatest area for nursery production in western United States. With annual production values greater than $1B, the Oregon nursery industry is three times the most valuable agricultural commodity in Oregon.

**Figure 1:**
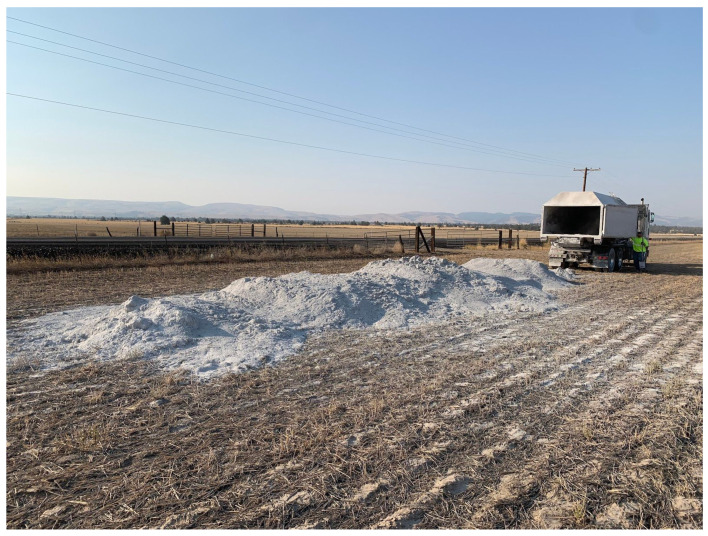
Delivery of tectonite material to wheat field in Maupin, OR, USA. Tectonite dust created as a byproduct of regional manufacturing. This field site was a replicated experiment assessing the impacts of different volumes of tectonite applications on the growth and yield of dryland farmed winter wheat.

As a result of the regional conventional and specialty crop industries, WSCP reached out to Oregon State University to conduct research on the potential for this novel, local by-product as an agricultural soil amendment. This research comprises a three-step process to investigate the potential of tectonite: (1) analysis of the physical properties of soilless substrates amended with tectonite; (2) a small-scale, short-term trial to determine any obvious phytotoxic effects that would negate the need for a larger trial; and (3) a farm-scale, full-season trial to determine the effects of tectonite-amended soil on grain yield (Figure 1).

## 2. Results

### 2.1. Chemical Property Analysis

Tectonite is a fine dust, with 94% of the tectonite particles measuring ≤0.149 mm in size, as determined by passing through a 100-mesh sieve. The water extract demonstrated the potential of tectonite to contribute salts, either as nutrients or metals, when placed in water. The resulting electrical conductivity was 1777 µs·cm^−1^. The primary water-extractable nutrients were calcium > sulfur > potassium > silicon. Extractable heavy metal concentrations were, on average, ≤0.265 µg·mL^−1^, with strontium being the highest at 2.835 µg·mL^−1^ (Table 1). The high pH (10.8) of tectonite during water extraction was notable because soilless mixes like bark and peat are naturally low pH, as are many soils in Oregon.

Further analysis was required to determine if compositional or extractable calcium, magnesium, and sulfur served as a lime or gypsum source when applied to soilless substrate or soil. Liming material analysis determined that half of the 20% calcium present in the product occurred as calcium carbonate, providing a calcium carbonate equivalence (CCE) of 17. Only 1.6% of the 0.3% magnesium in tectonite contributed to CCE as magnesium carbonate, even though MgCO₃’s acid-neutralizing power is greater than its calcium equivalent [12].

### 2.2. Physical Property Analysis

The physical property analysis of the bark mixes revealed that tectonite increased the total porosity (TP) compared to pure DFB, and at 30% amendment, the TP was not significantly different from the commercial PM. The air space (AS) in the bark mixes showed a significant difference only at the 10% tectonite blends. Container capacity (CC) was significantly increased in DFB blends with 20% and 30% tectonite amendment, with 30% amendment resembling the PM. The TP and CC of AP soils were unaffected by tectonite amendment. The AS decreased in AP soils amended with 20% or more tectonite. Adding tectonite to the JF soils increased TP and decreased AS. Tectonite also increased CC in the JF soils, with 20–30% amendment holding about 10% more water than the unamended soils (Table 2).

The physical properties analysis suggests that tectonite can be added to soilless and mineral substrates with different beneficial effects. The commercial blend sold is a mix of 70% fine DFB bark, 20% DFB mulch, and 10% pumice with a wetting agent (OBC, Inc., Canby, OR, USA). Compared to 100% DFB the commercial blend has significantly greater TP, and CC. Yet, by adding 30% tectonite the TP and CC were not significantly different between DFB and the commercial blend (Table 2). The physical analysis of the mineral soils from the Agency Plains (AP) and Juniper Flats (JF) areas revealed different responses to tectonite amendments. For the AP soil, only AS was significantly affected by tectonite amendments, with higher concentrations (20% and 30%) having decreased AS. Alternatively, JF soil TP and CC were increased even at 10% amendment and increasingly so with 30% tectonite. The different responses between the two mineral soils were somewhat surprising given that both AP and JF are in the Loam textural class (USDA-NRCS).

### 2.3. Plant Growth Trials

**Figure 2:**
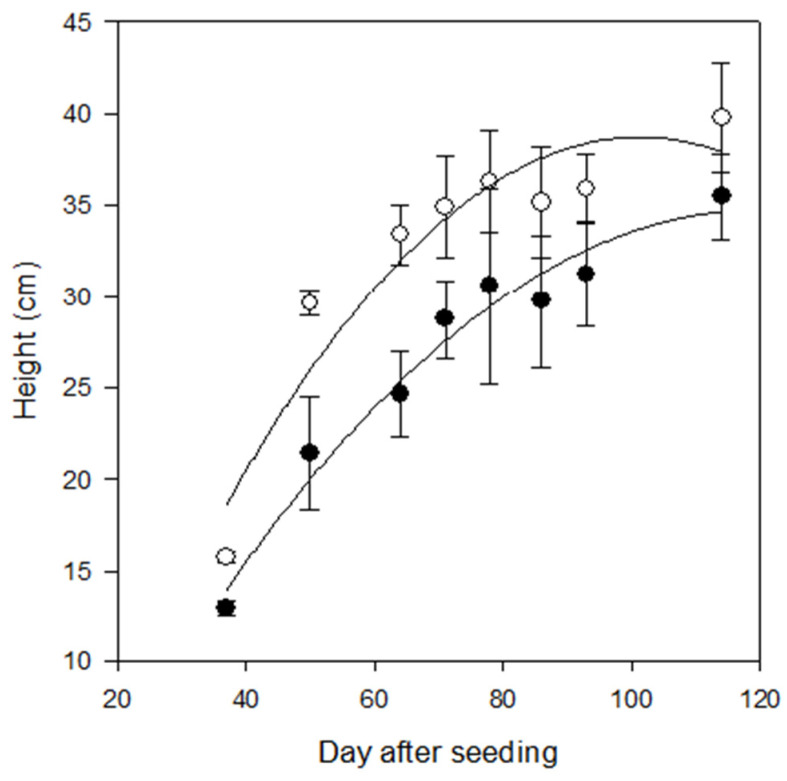
Wheat height measurement at a small-scale experiment conducted 1 June 2020–30 September 2020, at NWREC. Plant heights were first measured 14 days after germination (8 July 2020), and periodically throughout the summer. The black circles represent the means heights of plants from the control group (i.e., without tectonite), and white circles represent mean heights of plants growing in the tectonite group, with standard deviation error bars (n = 4). The results show consistently taller plants grown in the tectonite-amended plots *p* < 0.001.

**Figure 3:**
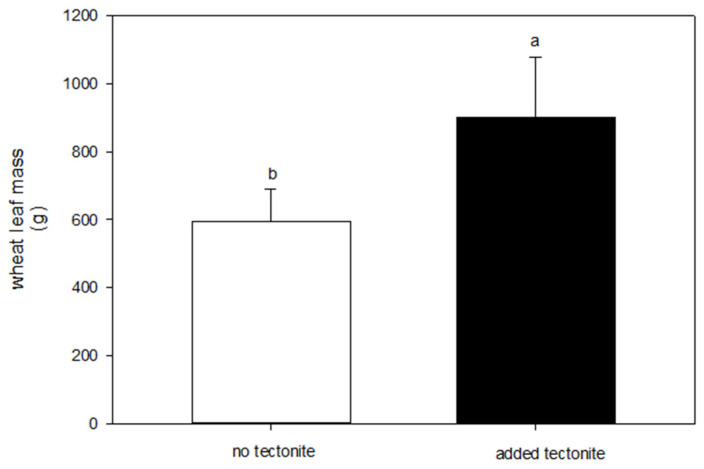
Dried plant biomass measurement from the small-scale experiment conducted 1 June 2020–30 September 2020 at NWREC. Wheat plants were clipped and dried to constant weight. The bar is the average (n = 4) mass per plot ± SD error bars. Means not sharing a letter are significantly different. The results show that plants grown on tectonite were larger than plants grown on unamended soil. The differences in the mean values among the treatment groups are greater than would be expected by chance (*p* = 0.022).

The small-scale field testing revealed promising growth responses by wheat when tectonite was applied to the Willamette Silt Loam soil at the NWREC. The wheat heights were consistently greater in the tectonite amended soil, a difference that manifested early in the season and was maintained throughout the trial (Figure 2). The disparity in the mean values among the two treatments (unamended soil vs. tectonite-amended soil) exceeded what would be expected by chance (*p* < 0.001). The multiple comparison procedure, to isolate which measurement days differed from the others revealed that the mean values were greater than would be expected by chance (*p* < 0.001) and therefore the effect of the treatment does not depend on time, e.g., day after start. In addition to height, the clipped biomass showed that the plants grown on the tectonite-amended soil were larger than the unamended soil (Figure 3).

**Figure 4:**
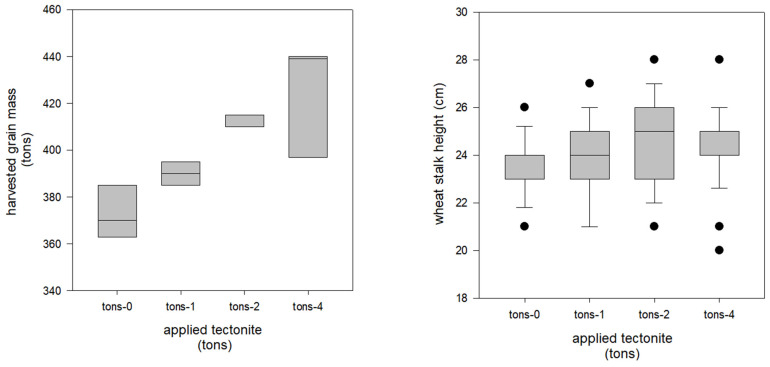
Wheat was harvested using a combine-tractor fitted with a GPS-integrated, load-cell-based yield monitor (HarvestMaster, Juniper Systems, Logan, UT, USA) to measure the mass of grain grown in each plot. Harvested grain mass (**Left**) was significantly improved by adding tectonite (*p* = 0.007). The height of the wheat stalks (**Right**) was not significantly different between the four treatment groups *p* > 0.05. The horizontal line within each box represents the median value. The error-bars extend to 1.5 times the inner quartile range, with dots marking any data point beyond this range.

Unlike the small-scale field trial the heights of the wheat were not different in the farm-scale experiment (Figure 4). We suspect that this was because of the much longer duration of the farm-scale growing season which was more than 2.5 times longer than the small-scale experiment. The farm-scale experiment did show a positive correlation between harvest yield and tectonite amendment. The results show that tectonite applications at two tons per acre (i.e., 4483.4 kg ha^−1^) and four tons per acre (i.e., 8966.8 kg ha^−1^) produced significantly more grain than the control plots that had zero tectonite (Figure 4). There was no significant difference between the average grain yield between 2× and 4× plots (411.67 ± 2.9 and 425.3 ± 24.5, respectively), suggesting that two tons per acre may sufficient if costs or supplies are limiting.

## 3. Discussion

For the soilless substrate the results showed that after adding 30% tectonite, the TP and CC were not significantly different between DFB and the commercial blend (Table 2). This discovery holds potential value in Oregon, where DFB serves as the primary soilless substrate in container crop production. The CC of a substrate is critical in all nursery production regions, particularly in Oregon, where dry summer conditions are normal, and insufficient substrate moisture can limit plant production, reduce yields, and impact profitability. Integrating tectonite into DFB could offer a sustainable solution for managing waste by-products. Growers using soilless substrates require multifunctional options that optimize growth and yield, are resource-efficient to minimize water waste [13], retain mineral nutrients effectively, and mitigate agrochemical loss. All of this must be achieved while ensuring regional availability and economic sustainability [14].

A potential challenge in linking tectonite production with nursery production is the physical distance (approximately 100 miles) between central Oregon, where tectonite is mined and milled, and the north Willamette Valley, the geographic center of the Oregon nursery industry. Previous research has highlighted that the cost of shipping and associated energy expenses can impede the upcycling of waste materials on tribal lands [15,16]. It’s noteworthy that a similar process currently exists, as pumice is mined and transported from central Oregon to the north Willamette Valley for use as an amendment in DFB mixes by the nursery industry [17]. Therefore, existing distribution networks could potentially be leveraged to transport tectonite waste to substrate manufacturers. A less energy-intensive solution could involve utilizing the tectonite waste locally.

Early growth and stand establishment is critically important for harvest and yield in the dryland summer fallow regions of the inland Pacific Northwest [18]. The small-scale trial represented an important progression beyond the soil physical analysis but was not conducted long enough to evaluate the effects on grain yield, which is a more critical metric for wheat. In the inland, Pacific Northwest, where dryland winter wheat is cultivated, the common practice for winter wheat is to plant in mid-to-late autumn. The timing is essential to capitalize on early season precipitation that will germinate the crop and allow for sufficient establishment before damaging winter cold temperatures. It has long been known that soil-moisture stress at any stage of growth can decrease wheat grain yield [19].

The physical property analysis of the field soil indicated that tectonite increased the water-holding capacity (i.e., CC) of the soil (Table 2), therefore the observed increases in yields could be associated with improved soil moisture characteristics. This hypothesis could be validated by additional studies that incorporate soil moisture sensors into long-term growth studies. Future studies might consider integrating soil moisture sensors for long-term assessment of water holding capacity [20,21], as well as remote sensing of stand establishment of wheat to compare early season growth and late season density.

Some potential mechanisms, by which improve soil health and crop yields include improving soil structure, increasing nutrient availability, and enhancing water-holding capacity. Beyond the benefits of enhanced soil moisture, there could be growth benefits associated with the additional calcium in tectonite. Tectonite contains 17% effective calcium carbonate and can function as a mild to moderate liming source when compared to commercial lime or dolomite products [22]. The Madras soil series, where our experiment was conducted, is moderately deep, well-drained mesic Aridic Argixerolls on upland terraces and plateaus that were formed in windblown deposits over volcaniclastic sediments from the Deschutes Formation (USDA-NRCS, 1998 [23]). These soils can have “high” magnesium levels that can lead to soil crusting, which reduces infiltration and therefore wheat stand establishment. Applying gypsum to the soil is the currently common practice by farmers in the region to reduce soil crusting and improve infiltration [24]. Gypsum is used because it is calcium-rich, dissolves at high pH, and is cheap and easy to use [25]. Like gypsum, the beneficial flocculating properties of tectonite may be responsible for the increased yield compared to the control plots. Soil flocculation can create larger pore spaces in the soil, which can improve water infiltration and retention, as well as air exchange in the root zone [24,25]. Additionally, the addition of calcium can help to reduce soil compaction and improve soil tilth, which can create a more hospitable environment for plant roots. If growers were to use tectonite as a substitute they would need to apply more material per acre since gypsum (23.2% Ca) has more Ca than tectonite. Another way that mineral amendments can improve soil health is through their ability to release nutrients into the soil. For example, gypsum contains sulfur, which can be converted by soil microbes into plant-available sulfate. Similarly, tectonite dust contains a range of minerals, including sulfur and potassium (Table 1) that can be slowly released into the soil over time, providing a sustained source of nutrients for plants.

The observed increase in water-holding capacity for certain mineral soils and soilless media, coupled with the measurable improvement in yields resulting from tectonite application, suggests that this waste product holds promising potential as an agricultural amendment. However, as with other novel soil amendments [26], there are potential risks associated with the use of tectonite dust. One concern is the potential for heavy metal contamination, as tectonite dust may contain trace amounts of heavy metals such as lead, cadmium, and arsenic (Table 1). To mitigate this risk, it is important to carefully source and test tectonite dust for heavy metal content before use, as well as monitor soil and plant tissue for any signs of contamination. Additional research on the use of tectonite dust as a soil amendment for edible crops is recommended to further understand its short and long-term impacts. Nevertheless, tectonite dust appears to exert a positive influence on enhancing the yield of commercial crops in central Oregon, potentially aiding in offsetting the increasing negative impacts of climate change, including reduced rainfall and higher temperatures [27,28].

The utilization of waste byproducts in agriculture can align with broader sustainability goals, such as reducing waste and mitigating greenhouse gas emissions. Leveraging waste products like tectonite dust allows farmers to decrease reliance on traditional inputs like synthetic fertilizers, which are often energy-intensive to produce and transport. Studies on wheat production in other parts of the world have highlighted that fertilizer inputs constitute one of the most energy-intensive aspects of production [29]. Therefore, locally sourced agricultural amendments like tectonite can play a role in optimizing agricultural operations. Furthermore, the use of waste products contributes to the reduction of waste in landfills, leading to a significant impact on reducing greenhouse gas emissions.

The positive outcomes of a novel, local source merit investigation due to potential advantages such as decreased transportation emissions, reduced landfill volume and costs, increased yields, and mitigation of dry climate challenges. This approach also holds the potential for economic stimulation for the Tribe by creating a new market for their raw materials. In terms of economic costs and benefits, the use of tectonite dust as a soil amendment may offer cost savings compared to synthetic fertilizers, especially for farmers located close to tectonite dust sources. Additionally, the use of tectonite dust may bring economic benefits to the local community by establishing a new market for waste products and reducing landfill volume and costs.

This waste reduction strategy aligns with the circular economy concept, described as a regenerative industrial system, which creates opportunities from the production of large amounts of waste to its elimination through the superior design of materials, products, systems, and business models [30]. Addressing the logistical costs of delivering tectonite dust to farms not proximate to WCSP will be a crucial factor in making tectonite dust more marketable to crop farmers throughout central Oregon. Overall, the use of tectonite dust as a soil amendment appears to offer both environmental and economic benefits compared to synthetic fertilizers, while also providing comparable or superior crop yields and soil health outcomes. However, careful sourcing and testing are imperative to mitigate potential risks associated with heavy metal contamination. Further research is needed to fully understand the short- and long-term impacts of tectonite dust use, but its potential benefits suggest that it is worth exploring as a viable option for promoting sustainable agriculture [31].

## 4. Materials and Methods

### 4.1. Chemical Property Analysis

Three analyses were provided of tectonite: percent of major elemental constituents provided by the WSCP, water extractable nutrients and metals (<0.45 um) determined by inductively coupled plasma optical emission spectroscopy (Agilent 5110 ICP-OES, Agilent Technologies, Inc., Santa Clara, CA, USA), and liming analysis performed by Brookside Laboratories (Bremen, OH, USA). Non-replicated chemical analysis were compared to infer effect of tectonite on electrochemical properties of soilless substrate and soil utilized herein.

### 4.2. Physical Property Analysis

On 1 April 2020, tectonite byproduct from WSCP was received at Oregon State University North Willamette Research and Extension Center in Aurora, OR, USA (NWREC) and physical properties were analyzed using the porometer method developed at North Carolina State University [32]. Briefly, the physical property analysis conventionally used in the US for soilless substrates consists of a series of steps comparing the mass of a known volume at different levels of moisture to characterize maximum water holding capacity (CC; *v*/*v*), minimum air space (AS; *v*/*v*), total porosity (TP; *v*/*v*), and bulk density (Db; g cm^−1^). Four substrates were selected for analysis: (1) a commercial potting mix (PM; 40% Coarse Douglas-fir [*Pseudotsuga menziesii* (Mirbel) Franco] bark (DFB), 40% (by vol.) fine DFB, 10% (by vol.) coconut coir, 10% (by vol.) perlite; (2) 100% (by vol.) aged DFB; (3) Madras loam mineral soil gathered near Agency Plains (AP) in Jefferson County, Oregon (44.69, −121.19); (4) a Maupin loam mineral soil gathered near Juniper Flats (JF) in Wasco County, OR, USA (GPS 45.17, −121.19). DFB is the most common soilless substrate used in container production in Oregon [33]. The PM was used as a “standard” to create a benchmark for physical properties that is acceptable in containerized specialty crop production. AP and JF were selected because these are common soil series in the farming regions in Oregon near WSCP. During the physical property test, DFB, AP, and JF were amended with 0%, 10%, 20%, and 30% tectonite (*v*/*v*). Substrate physical properties of PM, including CC, AS, TP, and Db were determined using three representative samples of each substrate analyzed (n = 3). For each measurable output (i.e., AS, TP, CC, and Db), a one-way analysis of variance (ANOVA) was used to determine if there were differences between the control groups (0% tectonite) and the samples with added tectonite. The PM was included in DFB statistical analysis because PM is also Douglas-fir bark based. Where appropriate, a pairwise multiple comparison procedure (Holm–Sidak method) was used to compare the differences within groups at an overall significance level = 0.05. In addition, a single aliquot of tectonite was analytically compared to gypsum (CaSO_4_-2H_2_O) by an independent laboratory (Pratum Co-Op Agronomy, Madras, OR, USA).

### 4.3. Plant Growth Trials

On 1 June 2020, a small-scale trial was initiated at NWREC (GPS 45.28, −122.75) to evaluate the effect of tectonite amendment on the growth of *Triticum aestivum* L. in a Willamette silt loam soil. The Ap horizon of the soil was tilled and cleared of vegetation prior to planting. The experimental field was divided into eight plots of 0.46 m^2^ (5 ft^2^) each, totaling 3.68 m^2^ (40 ft^2^), and randomly assigned to either a control group (no amendment) or a tectonite group. For the tectonite plots (n = 4), 0.28 m^3^ (10 ft^3^) of soil was removed and replaced with 0.28 m^3^ (10 ft^3^) of tectonite. The tectonite was mixed evenly with the remaining 0.94 m^3^ (34 ft^3^) of soil using two wheelbarrows. For the control plots (n = 4), the soil was turned over in the same way as the tectonite plots, without the addition of any tectonite. Each plot was trenched to a depth of 30 cm (1 ft), a width of 30 cm (1 ft), and a length of 1.52 m (5 ft) for a soil volume of 0.22 m^3^ (8 ft^3^) per plot. After mixing, the soils were emptied back into the trenched plots and smoothed flat with a rake. The trial was conducted over a period of 120 days. Prior to planting, the field was watered using a portable impact sprinkler until each plot was saturated. The following day, three rows, 20 cm apart, of wheat were hand seeded in 10 cm in-row spacing for each plot. Every week for the following eight weeks, wheat height (cm) was measured in three locations for each replication. The three within-plot height locations were selected in stratified random sampling by alternating within plot rows (1–3) and sections (north, middle, and south).

On 1 October 2020, a farm-scale experiment was initiated in a 1.204 ha agricultural region of Wasco County, Oregon, with a Maupin loam-type soil. The experiment consisted of twelve plots of 0.081 ha each, randomly assigned to four treatments: (1) 0 kg ha^−1^, (2) 2471 kg ha^−1^ (1 ton ac^−1^), (3) 4942 kg ha^−1^ (2 ton ac^−1^), and (4) 8960 kg ha^−1^ (4 ton ac^−1^), with three replicates per treatment. Tectonite was surface spread on the experimental plots using GPS with a lime spreader on the same day. On 20 October 2020, wheat was seeded after the first rainfall event before the onset of winter storms and grown for approximately 270 days with no additional fertilizer or supplemental water via irrigation. On 27 July 2021, the height (cm) of the dryland wheat was measured three times at three different locations (on the East side, in the middle, and on the West side) within each 0.081 ha plot. On 3 August 2021, the wheat was harvested using a combine-tractor fitted with a GPS-integrated, load-cell-based yield monitor (HarvestMaster, Juniper Systems, Logan, UT, USA) to measure the mass of grain grown in each plot. The aboveground biomass was hand clipped from each plot, placed in paper bags, and dried at 80 °C in a forced air oven for 48 h. The mass (g) of the wheat was weighed and recorded.

### 4.4. Statistical Analysis

Analysis of variance (ANOVA) was used to determine differences between the control groups (0% tectonite) and the plots with added tectonite. If necessary, a pairwise multiple comparison procedure (Holm–Sidak method) was used to compare the differences within groups at an overall significance level of 0.05.

## 5. Conclusions

Climate-ready agriculture demands a holistic approach encompassing research, education, and extension activities to prepare the agricultural workforce for the challenges posed by climate change. This study contributes to this imperative by exploring the potential of tectonite dust as a sustainable soil amendment in central Oregon. As we adapt to climate change, it becomes crucial to not only mitigate its adverse effects but also identify new opportunities for sustainable agricultural practices.

In the quest for improved soil health and enhanced crop yields, our research has shed light on the multifaceted mechanisms that tectonite dust introduces, such as improvements in soil structure, increased nutrient availability, and enhanced water-holding capacity. The additional calcium content in tectonite offers a unique dimension to potential growth advantages. Focusing our experimentation on the Madras soil series, characterized by moderately deep, well-drained mesic Aridic Argixerolls situated on upland terraces and plateaus, provides insights tailored to the regional context.

Encouraging additional research on the application of tectonite dust as a soil amendment, particularly for edible crops, is advisable to gain a comprehensive understanding of its short- and long-term effects. While our preliminary observations suggest positive impacts on crop yields in central Oregon, further investigations will contribute to a nuanced understanding of tectonite’s potential benefits in mitigating climate change impacts, including altered rainfall patterns and extreme temperatures.

The incorporation of waste byproducts into agriculture represents a significant stride toward broader sustainability goals, including waste reduction and the mitigation of greenhouse gas emissions. Tectonite dust emerges as a promising alternative, enabling farmers to reduce reliance on energy-intensive synthetic fertilizers. The economic considerations are noteworthy, with potential cost savings for farmers near tectonite dust sources and the creation of a new market for waste products, thereby lowering landfill volume and associated costs.

In conclusion, this study explores the novel application of tectonite dust in agriculture, revealing its potential as a sustainable soil amendment with economic and environmental benefits. As we navigate the challenges of climate change, the findings contribute to the broader understanding of climate-ready agriculture and underscore the importance of region-specific investigations for sustainable practices.

## Figures and Tables

**Table 1:** Type 1 water (185 mL) extractable metals (µg·mL^−1^) from 80 g of tectonite (n = 3). Metals were determined by inductively coupled plasma spectroscopy of a 20 mL–0.45 um filtered aliquot of extractant. Extract pH was 10.8 and electrical conductivity 1777 µs·cm^−1^. Below detection limit is abbreviated as BDL, with detection limit for Pb being < 0.002 and Be, Cd, Co being < 0.004.

Metal	Mean		Std Dev	Metal	Mean		Std Dev
**Al**	0.036	±	0.003	**Mn**	0.015	±	0.018
**As**	0.025	±	0.004	**Mo**	0.021	±	0.001
**B**	0.153	±	0.025	**Na**	70.470	±	5.002
**Ba**	0.123	±	0.03	**Ni**	0.007	±	0.009
**Be**	BDL	±	BDL	**P**	0.079	±	0.029
**Ca**	772.133	±	48.596	**Pb**	BDL	±	BDL
**Cd**	BDL	±	BDL	**S**	506.700	±	33.108
**Co**	BDL	±	BDL	**Sb**	0.011	±	0
**Cr**	0.150	±	0.008	**Se**	0.025	±	0.008
**Cu**	0.011	±	0.005	**Si**	40.640	±	2.694
**Fe**	0.011	±	0.005	**Sr**	2.835	±	0.158
**K**	45.803	±	11.46	**Tl**	0.011	±	0.002
**Li**	0.004	±	0	**V**	0.262	±	0.012
**Mg**	0.394	±	0.06	**Zn**	0.038	±	0.023

**Table 2:** Physical properties of 100% Douglas-fir bark (DFB) and 100% mineral soils from Agency Plains, Oregon (AP) and Juniper Flats, Oregon (JF) amended (by volume) 10%, 20%, and 30% tectonite mineral dust. Differences between treatment groups for the physical properties and two field trials were compared with analysis of variance (ANOVA) and pair-wise comparison with a Tukey test. Reported means not sharing any letter are significantly different than other means within each substrate at the level of significance *p* < 0.05. Total porosity is equal to the container capacity + air space. Air space is the column of water drained from the sample ÷ volume of the sample. Container capacity is (wet weight—oven dry weight) ÷ volume of the sample. Bulk density after forced air drying at 105 °C (221 °F) for 48 h.

Substrate	Sample Size	Total Porosity (avg. % vol)	±		Air Space (avg. % vol)	±		Container Capacity (avg. % vol)	±		Bulk Density (avg. g cm^−3^)	±	
Commercial Blend	12	71.6	5.7	a	17.9	4.9	b	53.7	1.7	a	0.22	0.01	d
100% DFB	12	48.9	11	c	16.2	10	b	32.7	6.7	c	0.21	0.01	e
DFB 10% tectonite	12	61.2	7.9	b	27.0	6.7	a	34.1	4.1	c	0.24	0.01	c
DFB 20% tectonite	12	60.8	1.9	b	14.5	3.5	b	46.3	2.8	b	0.29	0.01	b
DFB 30% tectonite	12	66.4	3	ab	16.4	3.3	b	50.0	2.3	ab	0.34	0.01	a
AP soil	12	53.7	3	a	6.3	0.84	a	47.4	2.3	a	1.05	0.01	a
AP 10% tectonite	12	56.9	1.5	a	6.9	0.49	a	50	1.6	a	1.06	0.01	a
AP 20% tectonite	12	53.5	3.9	a	5.3	0.78	b	48.2	3.5	a	1.1	0.04	b
AP 30% tectonite	12	53.4	4.8	a	5.0	1.3	b	48.3	4.2	a	1.04	0.03	a
JF soil	12	53.4	1.4	c	13.6	1.1	a	39.8	1.7	c	0.97	0.01	a
JF 10% tectonite	12	56.5	1.1	b	8.0	1	b	48.3	1.4	b	0.96	0.01	a
JF 20% tectonite	12	58.5	1.1	a	7.4	0.87	b	51.1	1.1	a	0.94	0.03	b
JF 30% tectonite	12	57.2	1.7	b	6.4	0.98	c	50.8	1.3	a	0.95	0.01	ab

## Data Availability

Data are contained within the article.
